# Exploring the risk association between psoriasis and chronic obstructive pulmonary disease, and asthma using the NHIS database

**DOI:** 10.1371/journal.pone.0342015

**Published:** 2026-02-13

**Authors:** Mingyu Liu, Shuming Ji, Pengxin Ye, Min Gong, Qilin Liu

**Affiliations:** 1 Department of Equipment, West China Hospital of Sichuan University, Chengdu, China; 2 Department of Clinical Research Management, West China Hospital of Sichuan University, Chengdu, China; 3 Department of Equipment, West China Hospital of Sichuan University, Chengdu, China; Istituto Dermopatico dell'Immacolata (IDI)-IRCCS, ITALY

## Abstract

**Background:**

Asthma (AS) and chronic obstructive pulmonary disease (COPD) are prevalent chronic airway disorders with shared inflammatory pathways. Considering the systemic inflammatory nature of psoriasis, this study utilized the National Health Interview Survey (NHIS) to investigate its potential association with COPD and AS.

**Methods:**

Data from the 2023 NHIS were analyzed, with participants selected based on specific inclusion criteria. A baseline table was constructed, and multivariate logistic regression models, along with risk stratification analysis, were employed to examine the correlations between psoriasis and COPD and AS. Receiver operating characteristic (ROC) analysis was conducted to assess the predictive value for both conditions.

**Results:**

A total of 27,106 participants were included in the study. Significant differences were observed in the baseline characteristics of psoriasis, age, race, region, gender, education, marital status, employment, income, smoking habits, health status, mental health, disability, heart attack history, BMI, prediabetes, cancer, hypertension, hypercholesterolemia, arthritis, coronary heart disease, stroke, health insurance, anxiety, and depression (*P* < 0.001). Multivariate logistic regression revealed a strong association between psoriasis and COPD (model 1: OR 2.63, 95% CI: 2.13–3.23, *P* < 0.001; model 2: OR 2.43, 95% CI: 1.95–2.99, *P* < 0.001; model 3: OR 1.54, 95% CI: 1.21–1.96, *P* < 0.001). A similar association was found between psoriasis and AS (model 1: OR 1.68, 95% CI: 1.42–1.97, *P* < 0.001; model 2: OR 1.74, 95% CI: 1.47–2.05, *P* < 0.001; model 3: OR 1.32, 95% CI: 1.11–1.56, *P* < 0.01). The ROC analysis based on model 3 demonstrated substantial predictive power of psoriasis for COPD, with an AUC of 0.881.

**Conclusion:**

Psoriasis was identified to have a strong association with COPD and AS, which provided valuable insights into understanding the pathogenesis of these diseases.

## 1. Introduction

Chronic obstructive pulmonary disease (COPD) and asthma (AS) are highly prevalent chronic respiratory diseases worldwide. Due to their distinct pathophysiological mechanisms and clinical manifestations, they have long been subjects of extensive attention in clinical practice and scientific research. COPD ranks as the second most common respiratory disease globally, characterized by persistent airflow limitation primarily associated with oxidative damage and airway remodeling triggered by exposure to tobacco smoke and harmful particulate matter [1,2]. AS is characterized by reversible airflow limitation, increased mucus secretion, and airway hyperresponsiveness, often triggered by allergens such as pollen or cold air [3]. There are also differences in the clinical treatment methods of the two: the current COPD treatment is mainly oxygen therapy, non-invasive ventilation, pulmonary rehabilitation and bronchodilators (such as LAMA/LABA) [4,5]; Conventional clinical treatment of AS still focuses on alleviating bronchial contraction [6]. However, despite the differences between the two, it is found in clinical practice that COPD and asthma often overlap (such as chronic cough, wheezing), and comorbidities are particularly prominent in smokers or obese people [7].This shows that although COPD and AS are significantly different, the overlapping clinical symptoms and comorbidities of the two highlight the necessity of exploring their potential common drivers.

Psoriasis is a chronic systemic inflammatory disease, and its onset is closely related to the activation of Th1/Th17 cells and the inflammatory response mediated by cytokines such as IFN-γ, TNF-α, and IL-17 [8]. These inflammatory pathways also happen to be involved in the progression of COPD and AS [9,10]. Existing studies have initially suggested an association between psoriasis and respiratory diseases, such as the NHANES 2009–2014 data showing a 67% increased risk of AS in patients with psoriasis [11]. However, these studies focus more on single airway diseases, and lack a joint analysis of two representative chronic airway diseases, COPD and AS, and it is difficult to fully reveal the correlation pattern of psoriasis and different types of airway diseases.

The National Health Interview Survey (NHIS) is an annual health survey conducted by the National Center for Health Statistics (NCHS) under the Centers for Disease Control and Prevention (CDC). As an authoritative source of information reflecting the health and well-being of the U.S. population, the survey covers areas including demographic information, health status, healthcare utilization, health insurance coverage, disability status, and work-related health issues [12,13]. Therefore, this study utilizes the 2023 NHIS database to systematically evaluate the association between psoriasis and COPD/AS through a multivariable logistic regression model and risk stratification analysis, exploring its predictive value to provide new insights for managing chronic airway diseases.

## 2. Materials and methods

### 2.1. Data acquisition

**Fig 1:**
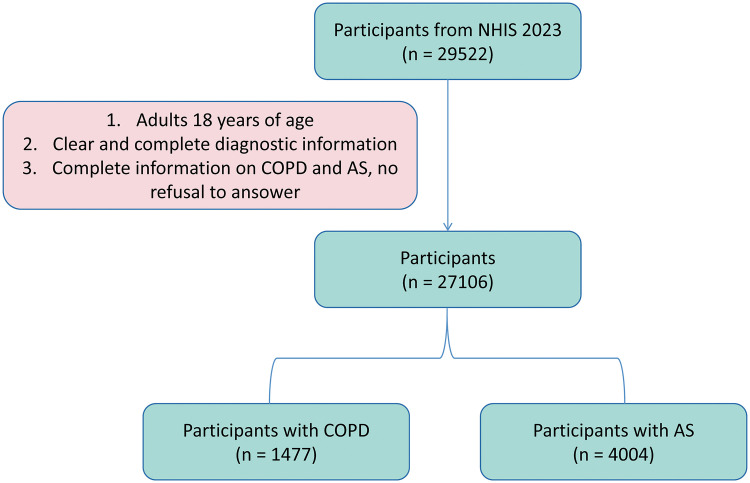
Process diagram for inclusion of research subjects.

The NHIS (https://www.cdc.gov/nchs/nhis/index.html) is an annual cross-sectional survey that collects health-related data from a nationally representative sample of civilian, non-institutionalized residents in the United States. In this study, COPD and AS were considered the outcome, with psoriasis as the exposure factors. Covariates such as sociodemographic characteristics, health status, comorbidities, and insurance coverage were also included in the analysis. A total of 29,522 participants from the 2023 NHIS dataset were initially considered. Inclusion criteria were as follows: 1) Adults aged 18 years or older; 2) Participants who responded with “Yes” and provided clear and complete diagnostic information; 3) Participants who completed all covariate information and did not refuse to answer or indicate responses as “unknown”. To further ensure no missing values in all analytical variables and guarantee data quality, during the data preprocessing stage, this study used the complete case analysis method to handle missing data. Specifically, samples with any missing values (NA) in the dataset were excluded, and only complete records with no missing values in all variables were retained. To further ensure the absence of missing values in all analytical variables and guarantee data quality, this study employed a missing proportion analysis method to handle missing data during the data preprocessing stage. Finally, after excluding samples with missing values and merging data of asthma and COPD cases, 27,106 eligible participants were identified and included in subsequent analyses (Fig 1).

### 2.2. Definition of outcome

Data for 2023, along with the Sample Adult Interview, were selected from the NHIS database. The codebook was reviewed, and COPDEV_A was queried: “Has a doctor or healthcare professional ever diagnosed you with COPD, emphysema, or chronic bronchitis?” Participants who answered “Yes” were categorized as having COPD, while those who responded “No” were classified as not having COPD. Similarly, the codebook was queried for ASEV_A: “Has a doctor or healthcare professional ever diagnosed you with asthma?” Participants who answered “Yes” were classified as having AS, while those who answered “No” were classified as not having AS.

### 2.3. Definition of exposure factor

To identify participants with psoriasis, the 2023 data and Sample Adult Interview were selected from the NHIS database. The codebook was accessed, and PSOREV_A was searched: “Have you ever been told by a doctor or other health professional that you had psoriasis?” Participants answering “Yes” were classified as having psoriasis, while those answering “No” were classified as not having psoriasis.

### 2.4. Definition of covariates

Several covariates were considered in this study, including age, race, region, gender, education, marital status, employment, income, smoking, health status, mental health, disability, heart attack, body mass index (BMI), prediabetes, cancer, hypertension, high cholesterol, arthritis, coronary heart disease, stroke, health insurance, anxiety, and depression [12,14–16]. Age was divided into five groups: ≥ 65, 50–64, 40–49, 18–29, and 30–39 years. Race was categorized into three groups: Hispanic (all other groups), Hispanic (Mexican/Mexican American), and Not Hispanic. Gender was divided into two categories: male and female. Education was classified into three categories: more than high school graduate, high school graduate, and less than high school graduate. Marital status was categorized into three groups: living with a partner, married, and neither. Income was divided into three categories: middle and high income, nearly poor, and poor. Smoking status was divided into four groups: every day, former smoker, never smoked, and some days. Region was categorized into four groups: West, Midwest, South, and Northeast. Health status was categorized into five groups: excellent, fair, good, poor, and very good. BMI was divided into four groups: healthy weight, obese, overweight, and underweight. Other covariates, such as employment, mental health, disability, heart attack, prediabetes, cancer, hypertension, high cholesterol, arthritis, coronary heart disease, stroke, health insurance, anxiety, and depression, were grouped as “Yes” or “No.” The covariate identifiers are provided in S1 Table.

### 2.5. Construction of the baseline table and risk association analysis

Chi-square test and t-test were used to compare differences in exposure factors and covariates among the control, COPD, AS, and combined COPD and AS groups. A baseline table was constructed using the tableone package (v 0.13.2) with a significance level set at *P* < 0.05 [17]. To examine the relationship between the exposure factors and outcomes, multivariate logistic regression models were developed. Model 1 assessed the association between exposure factors and outcomes without adjusting for covariates. Model 2 incorporated race, age, and gender as adjustments to Model 1. Model 3 further included region, education, marital status, employment, income, smoking, health status, mental health, disability, heart attack, BMI, prediabetes, cancer, hypertension, high cholesterol, arthritis, coronary heart disease, stroke, health insurance, anxiety, and depression. Odds ratios (OR), 95% confidence intervals (CI), and *P*-values were calculated to determine the strength of the associations. Logistic regression was performed separately for COPD and AS as outcomes. Meanwhile, goodness-of-fit tests and explanatory power index analyses were conducted for the three models, with the calculation of each model’s akaike information criterion (AIC) value, Nagelkerke R^2^ value, area under the curve (AUC) value, and Hosmer-Lemeshow (HL) test P-value.

To avoid potential bias caused by multicollinearity among covariates in Model 3 and ensure the reliability of regression results, we conducted a multicollinearity diagnosis. We used the vif function in the R package “car” (v 3.1.2) (https://socialsciences.mcmaster.ca/jfox/Books/Companion/) to calculate the Variance Inflation Factor (VIF) for Model 3 of both Asthma and COPD, which was applied to diagnose multicollinearity. Among the diagnostic criteria, VIF < 5 indicated no significant multicollinearity, VIF > 5 indicated strong multicollinearity, and VIF > 10 was considered severe multicollinearity.

To explore the influence of different covariates and exposure factors on COPD and AS across subgroups, risk stratification analysis was conducted based on Model 3. First, the regression coefficients of each independent variable (including psoriasis and all covariates in Model 3) were extracted using the glmnet package (v 4.4.1) with *P* < 0.05 [18]. This coefficient was essentially the log-odds, reflecting the logarithmic effect of the variable on the risk of the outcome (COPD or AS). Second, the extracted log-odds were exponentiated to yield the corresponding odds ratio (OR), which intuitively demonstrated the magnitude of the variable’s impact on the outcome risk. Subsequently, the 95% confidence interval (95% CI) for the log-odds was calculated using the confint() function in base R, and this interval was exponentiated to obtain the 95% CI for the OR, so as to verify the statistical reliability of the risk estimation. Finally, the forestplot package (v 3.1.1) was used to visualize the OR values and 95% CIs of all variables through forest plots [14].

### 2.6. Smooth curve fitting and receiver operating characteristic (ROC) analysis

To assess the nonlinear relationship between psoriasis and COPD, as well as between psoriasis and AS, Model 1 was used for adjustments, and the LOESS method was applied to plot smooth curves to validate these relationships.

In addition, to evaluate the predictive value of psoriasis for COPD and AS, t this study conducted receiver operating characteristic (ROC) analysis based on Model 3, which included all pre-specified covariates and exposure factors. The specific procedures were as follows: Using the pROC package (v 6.0.93) [15] in R software, we implemented the roc() function with the predicted probabilities from Model 3 as the independent variable and the disease status (COPD/AS) as the dependent variable. This generated ROC curves and calculated the area under the curve (AUC). An AUC value exceeding 0.7 indicated that the model demonstrated satisfactory predictive performance for the outcomes.

### 2.7. Statistical analysis

A two-tailed *P*-value < 0.05 was considered statistically significant for all analyses in this study. Specific statistical tests were applied as follows: for the construction of the baseline table, the chi-square test was used to compare differences in exposure factors and covariates among the control group, COPD group, AS group, and the group with both COPD and AS; for the multivariate logistic regression models and risk stratification analysis based on the glmnet package, the Wald test was used to calculate two-tailed P-values, so as to assess the statistical significance of the associations between variables and the effects of each variable in subgroup analyses.

All bioinformatics analyses and result visualizations were performed using R software (version 4.2.2), which covered the entire analytical workflow, including: data import and cleaning of the 2023 NHIS dataset; construction of the baseline table via the tableone package (v 0.13.2); performance of multivariate logistic regression analysis; conduction of risk stratification analysis via the glmnet package (v 4.4.1); implementation of ROC analysis via the pROC package (v 6.0.93); generation of forest plots via the forestplot package (v 3.1.1); and creation of smooth curves via the LOESS method.

Furthermore, a statistical power analysis was performed for all samples to evaluate the ability of the current sample size to detect the observed effect sizes in the research on asthma and COPD. This analysis employed a post-hoc power analysis approach, encompassing 25 effect levels across 13 predictors.

## 3. Results

### 3.1. Psoriasis showed a highly significant difference among the control group, COPD group, AS group, and the group with both COPD and AS

After excluding samples with missing values, 27,127 participants were included, accounting for 91.9% of the original sample size. All variables maintained low missing proportions, indicating good data quality. Specifically, the missing rates of region, race, disability status, and gender were 0%. Employment status had the highest missing proportion at 4.3% (S1A Fig). Subsequently, data of asthma and COPD cases were merged, and a total of 27,106 participants were included in subsequent analyses.

In this study, 27,106 participants were enrolled and divided into a control group (22,213 cases), a COPD group (889 cases), an asthma group (3,416 cases), and a COPD combined with AS group (588 cases). Significant differences (P < 0.05) were observed among the four groups in terms of demographic characteristics and covariate distributions. The COPD group predominantly consisted of elderly individuals (67.4% aged ≥65 years), females (53.7%), and non-Hispanic whites (96.1%), with significantly higher prevalence rates of hypertension, hypercholesterolemia, and arthritis compared to the other groups (P < 0.05). The asthma group had a wider age distribution (24.7% aged ≥ 65 years) and a higher proportion of obesity (41.3%), suggesting that metabolic factors might be involved in disease development. Notably, the prevalence of psoriasis in the COPD combined with AS group (9.4%) was higher than that in the control group (2.8%), initially indicating a potential association with respiratory diseases (Table 1).

**Table 1:** Baseline feature analysis of research subjects.

		Stratified by COPD: Asthma				
	level	control	COPD	AS	COPD and AS	p
n		22213	889	3416	588	
Age (%)	≥ 65	7354 (33.1)	599 (67.4)	844 (24.7)	315 (53.6)	< 0.001
	18-29	2647 (11.9)	9 (1.0)	648 (19.0)	5 (0.9)	
	30-39	3603 (16.2)	17 (1.9)	666 (19.5)	24 (4.1)	
	40-49	3208 (14.4)	44 (4.9)	500 (14.6)	46 (7.8)	
	50-64	5401 (24.3)	220 (24.7)	758 (22.2)	198 (33.7)	
Gender (%)	Female	11714 (52.7)	477 (53.7)	2016 (59.0)	386 (65.6)	< 0.001
	Male	10499 (47.3)	412 (46.3)	1400 (41.0)	202 (34.4)	
Race (%)	Hispanic (all other groups)	1472 (6.6)	17 (1.9)	252 (7.4)	27 (4.6)	< 0.001
	Hispanic (Mexican/Mexican American)	1947 (8.8)	18 (2.0)	221 (6.5)	18 (3.1)	
	Not Hispanic	18794 (84.6)	854 (96.1)	2943 (86.2)	543 (92.3)	
Education (%)	High School Grad	5540 (24.9)	328 (36.9)	796 (23.3)	167 (28.4)	< 0.001
	Less Than High School Grad	1819 (8.2)	143 (16.1)	215 (6.3)	92 (15.6)	
	More Than High School Grad	14854 (66.9)	418 (47.0)	2405 (70.4)	329 (56.0)	
Marital_Status (%)	Living with partner	1452 (6.5)	34 (3.8)	294 (8.6)	33 (5.6)	< 0.001
	Married	10588 (47.7)	300 (33.7)	1393 (40.8)	194 (33.0)	
	Neither	10173 (45.8)	555 (62.4)	1729 (50.6)	361 (61.4)	
Employment (%)	No	9445 (42.5)	721 (81.1)	1378 (40.3)	446 (75.9)	< 0.001
	Yes	12768 (57.5)	168 (18.9)	2038 (59.7)	142 (24.1)	
Income (%)	Middle and high income	16324 (73.5)	475 (53.4)	2410 (70.6)	269 (45.7)	< 0.001
	Nearly poor	3854 (17.4)	252 (28.3)	613 (17.9)	186 (31.6)	
	poor	2035 (9.2)	162 (18.2)	393 (11.5)	133 (22.6)	
Smoking (%)	every day	1587 (7.1)	254 (28.6)	250 (7.3)	126 (21.4)	< 0.001
	Former	5425 (24.4)	441 (49.6)	817 (23.9)	249 (42.3)	
	Never	14615 (65.8)	161 (18.1)	2256 (66.0)	183 (31.1)	
	some day	586 (2.6)	33 (3.7)	93 (2.7)	30 (5.1)	
Region (%)	Midwest	4873 (21.9)	229 (25.8)	744 (21.8)	130 (22.1)	< 0.001
	Northeast	3385 (15.2)	118 (13.3)	561 (16.4)	101 (17.2)	
	South	8300 (37.4)	373 (42.0)	1191 (34.9)	227 (38.6)	
	West	5655 (25.5)	169 (19.0)	920 (26.9)	130 (22.1)	
Health (%)	Excellent	5031 (22.6)	31 (3.5)	519 (15.2)	15 (2.6)	< 0.001
	Fair	2369 (10.7)	279 (31.4)	490 (14.3)	210 (35.7)	
	Good	6417 (28.9)	301 (33.9)	1085 (31.8)	167 (28.4)	
	Poor	552 (2.5)	154 (17.3)	138 (4.0)	120 (20.4)	
	Very Good	7844 (35.3)	124 (13.9)	1184 (34.7)	76 (12.9)	
Mental (%)	No	19550 (88.0)	758 (85.3)	2716 (79.5)	471 (80.1)	< 0.001
	Yes	2663 (12.0)	131 (14.7)	700 (20.5)	117 (19.9)	
Disability (%)	No	20317 (91.5)	597 (67.2)	3004 (87.9)	348 (59.2)	< 0.001
	Yes	1896 (8.5)	292 (32.8)	412 (12.1)	240 (40.8)	
BMI (%)	Healthy weight	7031 (31.7)	251 (28.2)	882 (25.8)	113 (19.2)	< 0.001
	Obese	7046 (31.7)	325 (36.6)	1411 (41.3)	288 (49.0)	
	Overweight	7811 (35.2)	269 (30.3)	1081 (31.6)	177 (30.1)	
	Underweight	325 (1.5)	44 (4.9)	42 (1.2)	10 (1.7)	
Heart_attack (%)	No	21509 (96.8)	744 (83.7)	3323 (97.3)	523 (88.9)	< 0.001
	Yes	704 (3.2)	145 (16.3)	93 (2.7)	65 (11.1)	
Prediabetes (%)	No	18590 (83.7)	624 (70.2)	2709 (79.3)	370 (62.9)	< 0.001
	Yes	3623 (16.3)	265 (29.8)	707 (20.7)	218 (37.1)	
Cancer (%)	No	19504 (87.8)	623 (70.1)	3029 (88.7)	451 (76.7)	< 0.001
	Yes	2709 (12.2)	266 (29.9)	387 (11.3)	137 (23.3)	
Hypertension (%)	No	14228 (64.1)	316 (35.5)	2154 (63.1)	201 (34.2)	< 0.001
	Yes	7985 (35.9)	573 (64.5)	1262 (36.9)	387 (65.8)	
High_cholesterol (%)	No	15203 (68.4)	395 (44.4)	2369 (69.4)	244 (41.5)	< 0.001
	Yes	7010 (31.6)	494 (55.6)	1047 (30.6)	344 (58.5)	
Arthritis (%)	No	17017 (76.6)	357 (40.2)	2438 (71.4)	178 (30.3)	< 0.001
	Yes	5196 (23.4)	532 (59.8)	978 (28.6)	410 (69.7)	
Coronary_heart_disease (%)	No	21009 (94.6)	678 (76.3)	3249 (95.1)	474 (80.6)	< 0.001
	Yes	1204 (5.4)	211 (23.7)	167 (4.9)	114 (19.4)	
Stroke (%)	No	21492 (96.8)	793 (89.2)	3303 (96.7)	506 (86.1)	< 0.001
	Yes	721 (3.2)	96 (10.8)	113 (3.3)	82 (13.9)	
Health_Insurance (%)	No	20616 (92.8)	857 (96.4)	3251 (95.2)	569 (96.8)	< 0.001
	Yes	1597 (7.2)	32 (3.6)	165 (4.8)	19 (3.2)	
Anxiety (%)	No	18730 (84.3)	623 (70.1)	2407 (70.5)	331 (56.3)	< 0.001
	Yes	3483 (15.7)	266 (29.9)	1009 (29.5)	257 (43.7)	
Depression (%)	No	18508 (83.3)	591 (66.5)	2381 (69.7)	297 (50.5)	< 0.001
	Yes	3705 (16.7)	298 (33.5)	1035 (30.3)	291 (49.5)	
Psoriasis (%)	No	21591 (97.2)	834 (93.8)	3278 (96.0)	533 (90.6)	< 0.001
	Yes	622 (2.8)	55 (6.2)	138 (4.0)	55 (9.4)	

### 3.2. Psoriasis shows significant associations with COPD and AS

Multivariate logistic regression analysis demonstrated that psoriasis was significantly associated with COPD and AS) and the effect sizes remained statistically significant after adjusting for different covariates (Table 2). In Model 1, which did not adjust for any covariates, the association between psoriasis and COPD was the strongest (OR = 2.633, 95% CI: 2.130–3.225, P < 0.001). After incorporating demographic variables (Model 2) and all covariates in the full model (Model 3), the OR value decreased to 1.544 (95% CI: 1.210–1.956, P < 0.001), yet it still retained statistical significance. Furthermore, Model 3 exhibited a discriminative ability with an AUC of 0.881 and a model explanatory power (Nagelkerke R^2^) of 0.3293, indicating that the model could effectively distinguish between COPD cases and non-cases. Additionally, all models demonstrated good goodness-of-fit, as evidenced by the HL test with P > 0.05.

**Table 2:** Risk association analysis of psoriasis with COPD and AS.

Disease	Model	OR_95 CI	P_value	AIC	Nagelkerke_R2	AUC	HL_P
Asthma	Model 1 (Crude)	1.677 (1.421-1.971)	<0.001	22669	0.0023	0.509	1
Asthma	Model 2 (Partially adjusted)	1.741 (1.473-2.048)	<0.001	22455.8	0.017	0.576	0.0031
Asthma	Model 3 (Fully adjusted)	1.319 (1.109-1.561)	0.0015	21482.5	0.0827	0.67	0.5172
COPD	Model 1 (Crude)	2.633 (2.130-3.225)	<0.001	11403.6	0.0072	0.522	1
COPD	Model 2 (Partially adjusted)	2.426 (1.952-2.990)	<0.001	10441.2	0.1095	0.729	0.9596
COPD	Model 3 (Fully adjusted)	1.544 (1.210-1.956)	<0.001	8282.6	0.3293	0.881	0.1113

Similarly, in Model 3, the risk effect of psoriasis on asthma was an OR of 1.319 (95% CI: 1.109–1.561, P = 0.0015), indicating that although its impact was weaker than that on COPD, it still held clinical significance. From Model 1 to Model 3, the Nagelkerke R^2^ gradually increased from 0.0023 in Model 1 to 0.0827 in Model 3, and the discriminative ability (AUC) improved from nearly random 0.509 to acceptable 0.670. The goodness-of-fit test indicated that the fully adjusted model (Model 3) exhibited a good fit, as evidenced by the HL test with P > 0.05.

Additionally, the stability of Model 3 was validated through multicollinearity diagnosis. The results showed that in Model 3 corresponding to COPD and AS, the variance inflation factors (VIFs) of all covariates were less than 5, confirming the absence of significant multicollinearity among the covariates and ensuring the reliability of the regression results (S2 Table).

**Fig 2:**
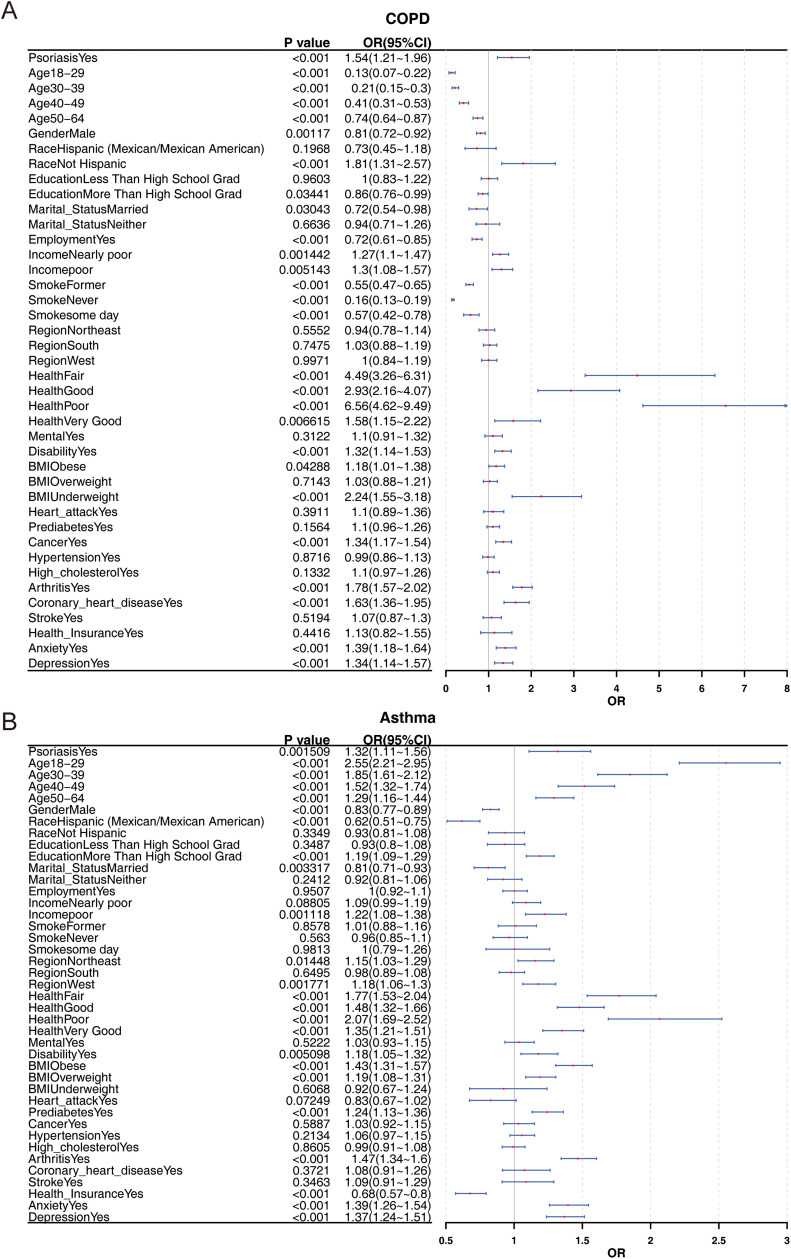
Risk correlation between COPD, AS, and psoriasis: (A) risk stratification analysis for psoriasis and COPD, and (B) risk stratification analysis for psoriasis and AS.

Risk stratification analysis further revealed a significant association between psoriasis and the onset of COPD (OR: 1.54, 95% CI: 1.21–1.96, *P* < 0.001). Additional risk factors for COPD included female gender, race (not Hispanic), income (nearly poor and poor), health status (fair, good, poor, and very good), disability, BMI (obese and underweight), cancer, arthritis, coronary heart disease, and anxiety (OR > 1, *P* < 0.05) (Fig 2A). Similarly, risk stratification analysis demonstrated a significant association between psoriasis and AS onset (OR: 1.32, 95% CI: 1.11–1.56, *P* < 0.01). Other risk factors for AS included age (18–29, 30–39, 40–49, and 50–64), education (more than high school graduate), income (poor), region (Northeast and West), health status (fair, good, poor, and very good), disability, BMI (obese and overweight), prediabetes, arthritis, anxiety, and depression (OR > 1, *P* < 0.05) (Fig 2B).

### 3.3. High statistical power

Post-hoc power analysis revealed that for asthma, 19 effects (e.g., covariates such as age, smoking status, and BMI) exhibited high statistical power (power ≥ 80%), 2 effects showed moderate power (power = 50–80%), and 4 effects had insufficient power (power < 50%). Specifically, the detection power for small effects with an OR of 1.2 reached 100%, while the power for moderate effects (OR = 1.5) and large effects (OR = 2.0) was also 100%. These results indicated that the sample size was sufficient for analyzing the vast majority of effects, enabling stable detection of their associations with asthma and minimizing the risk of type II error (missing true effects) (S1B Fig).

For COPD, the statistical power was even more robust: 24 effects demonstrated high statistical power (power ≥ 80%), with only 1 effect showing insufficient power and no effects classified as moderate power. The detection power for effects with an OR of 1.2 was 94.4%, and for OR values of 1.5 and 2.0, the power reached 100% (S1C Fig).

Collectively, the included sample size of 27,106 met the statistical power requirements for epidemiological studies.

### 3.4. Psoriasis had good predictive value for COPD

**Fig 3:**
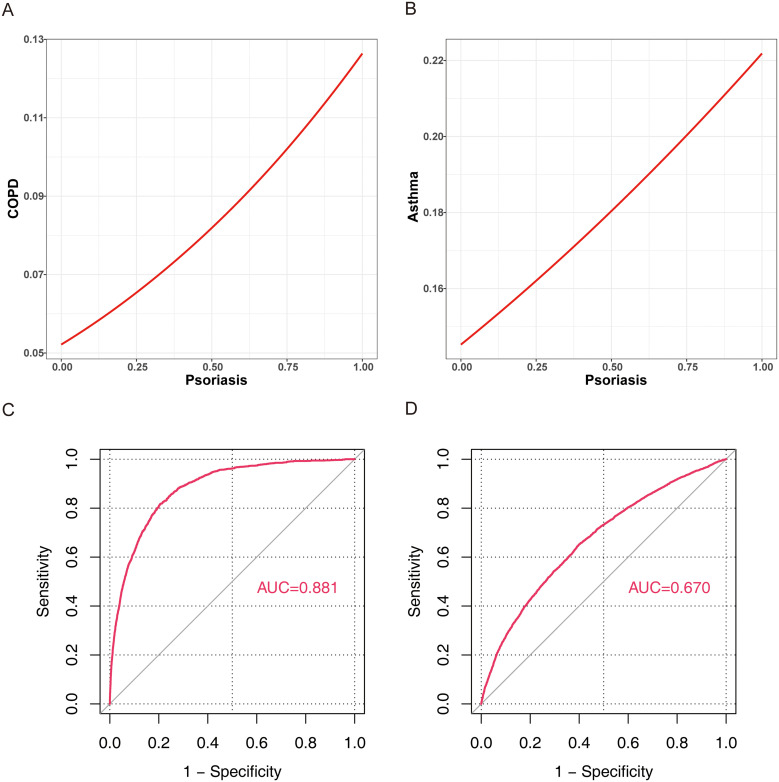
Correlation analysis of COPD, AS, and psoriasis: (A) correlation between psoriasis and COPD, (B) correlation between psoriasis and AS, (C) ROC curve of Model 3 for predicting COPD, and (D) ROC curve of Model 3 for predicting AS.

Psoriasis was positively correlated with the prevalence of both COPD and AS (Fig 3A, B). ROC analysis based on Model 3 revealed that this model had a substantial predictive value for COPD, with an AUC of 0.881 (Fig 3C). In contrast, the model demonstrated limited predictive value for AS, with an AUC of 0.670 (Fig 3D).

## 4. Discussion

COPD and AS are prevalent chronic diseases, and as the population grows, the number of individuals affected by these conditions continues to rise. Wu et al., in a meta-analysis, identified psoriasis as a risk factor for COPD [16]. Furthermore, Mateusz et al. demonstrated that childhood AS increases the likelihood of developing psoriasis [19]. Therefore, the aim of this study was to analyze data on COPD, AS, and related variables from the NHIS database. The data were filtered based on the baseline table, and a multivariate logistic regression model was developed to explore the influencing factors for both COPD and AS. The objective was to offer new insights and concepts for the prevention and treatment of these diseases.

After adjusting for multiple confounding factors including age, ethnicity, smoking status, and comorbidities, this study still observed a strong association between psoriasis and COPD. The ROC curve suggests the predictive power of psoriasis for COPD, suggesting that the two may be associated through underlying pathophysiological mechanisms. According to previous studies, the association between psoriasis and COPD may be achieved through inflammation. Psoriasis patients have abnormal proliferation and differentiation of skin keratinocytes, and recruited immune cells produce pro-inflammatory cytokines, which can reach the lungs through blood circulation [20]. Meanwhile, chronic inflammation characterized by immune cell infiltration also exists in the lungs of COPD patients [21–23]. In addition, the prevalence of COPD in psoriasis patients is higher than that in the general population, and the risk of COPPD increases with the severity of psoriasis [24]. The correlation analysis of this study showed a positive correlation between psoriasis and COPD. Therefore, psoriasis may to some extent promote changes in the pathological characteristics of COPD, and at the same time, the two diseases may have common risk factors or pathophysiological basis, thereby exhibiting a comorbidity tendency. This potential interaction pathway provides a starting point for elucidating the pathological association between psoriasis and COPD, suggesting that psoriasis can be included as a risk assessment indicator for COPD. It provides a theoretical basis for the development of COPD related monitoring and intervention strategies to regulate inflammation in psoriasis patients. However, further validation of related hypotheses is still needed.

In terms of the association between psoriasis and asthma, this study confirms a significant correlation between the two. Based on existing research, it is speculated that this association may be manifested through the synergistic effect of genetic susceptibility (such as sharing human leukocyte antigen (HLA) system susceptibility genes [25]) and common inflammatory pathways (both dominated by Th17 cell responses and sharing key inflammatory molecules such as IL-17 and IL-23 [26]). Specifically, the sustained release of pro-inflammatory cytokines in psoriasis patients can reach the lungs through blood circulation, exacerbating airway barrier damage, recruiting inflammatory cells, and inducing airway hyperresponsiveness [4,27]. suggesting that it may affect the occurrence and development of asthma. However, ROC analysis shows that psoriasis has a good predictive power for COPD, but relatively poor predictive power for asthma. This difference may be due to the closer interaction between the chronic inflammatory pathways of psoriasis and COPD, and the more predictable progression of COPD inflammation related lung function damage [9,28];The onset of asthma is influenced by multiple factors such as environmental allergens [3], which weakens the synergistic predictive effect of psoriasis and covariates. This difference indicates that psoriasis has disease-specific predictive value for chronic airway diseases, which can provide differentiated references for clinical risk stratification and early screening of COPD and asthma.

This study indicates that, in addition to psoriasis, multiple factors including age, ethnicity, geographic region, gender, educational attainment, marital status, employment status, income, smoking history, health status, and psychological state are associated with COPD and asthma. Among these, smoking and anxiety/depression are classic risk factors for COPD and asthma. Specifically, smoking induces increased neutrophil infiltration and heightened mucus secretion in airway epithelial cells of COPD patients, accelerating airway remodeling and irreversible airflow limitation [29]. For asthma, smoking may compromise the integrity of the airway mucosal barrier, increasing the likelihood of sensitization to external allergens [30]. The impact of adverse psychological states such as anxiety and depression on COPD and asthma primarily manifests at the neuroendocrine level. Prolonged anxiety and depression activate the hypothalamic-pituitary-adrenal (HPA) axis and sympathetic nervous system, leading to abnormal cortisol secretion rhythms [31,32], thereby weakening the body’s ability to suppress chronic inflammationand indirectly promoting the progression of both diseases [33]. Although this study confirmed the association between psoriasis and COPD-asthma after adjusting for these covariates, the presence of moderating factors such as smoking and psychological status in the COPD-asthma association suggests that these factors may interact with psoriasis. Based on the above findings, more targeted population management plans can be developed in clinical practice. Firstly, it is recommended to include early screening for COPD and asthma in the routine follow-up pathway for psoriasis patients, especially those with moderate to severe psoriasis. Regular testing of lung ventilation function and bronchial provocation or relaxation tests should be conducted to initiate early management of respiratory comorbidities and achieve early intervention for comorbidities; Secondly, for patients diagnosed with comorbidities (psoriasis combined with COPD/asthma), a multidisciplinary team (MDT) follow-up model consisting of dermatology, respiratory medicine, psychology, and general practice will be established: dermatology will monitor changes in psoriasis condition and adjust anti-inflammatory treatment, respiratory medicine will dynamically evaluate lung function and airway inflammation, and stepwise optimize bronchodilators and/or inhaled hormone regimens, psychological medicine will screen and intervene in anxiety/depression states through scales, and general practice will manage patients’ lifestyles (such as smoking cessation, nutrition, and vaccine guidance), and conduct regular multidisciplinary joint evaluations to ensure the integrity and coherence of treatment plans. Therefore, it is recommended that clinical management integrate the above risk factors into quantifiable intervention targets, block disease progression pathways through multidimensional risk regulation strategies, and achieve a full process management upgrade from “symptom control” to “trajectory rewriting”.

This study investigated the association between psoriasis and COPD/AS using the 2023 NHIS database. Although core findings were validated through multiple regression models, risk stratification, and ROC analysis, three methodological limitations remain: First, the initial analysis did not employ a sampling design incorporating stratification, clustering, and sampling weights, potentially introducing bias in effect estimates and necessitating caution in extrapolating conclusions. Second, the NHIS database, designed to cover broad topics, offers limited response options per question, making it difficult to obtain detailed information on COPD and asthma diagnosis/treatment or disease subtype data, thus failing to meet the requirements for in-depth research. Thirdly, the outcome measures of this study are based on retrospective data of ‘previously diagnosed’, which cannot clearly determine the chronological order of psoriasis with COPD and AS, and there is a limitation of lack of temporal correlation. Therefore, the results of this study reveal a disease association rather than causal effects. To address these limitations, future efforts will advance in three directions: First, incorporate NHIS sampling weights, stratification, and cluster identifiers into statistical models to correct for sampling design bias. Second, integrate structured clinical indicators from electronic health record (EHR) systems, such as pulmonary function test results and acute exacerbation hospitalization records. Concurrently, a multicenter prospective cohort study will be conducted to systematically collect serum inflammatory markers (e.g., IL-17, IL-23) and respiratory microbiome data. This dual approach of methodological correction and data supplementation will enhance result reliability, providing more rigorous evidence-based support for elucidating the association mechanisms between psoriasis and chronic airway diseases, as well as enabling precise risk stratification. Finally, regarding the causal relationship between exposure and outcome, prospective cohort studies or Mendelian randomization designs were used to further validate its directionality.

## 5. Conclusion

This study, based on the 2023 NHIS database, employed methods such as multivariate logistic regression to investigate the association between psoriasis and COPD/AS, as well as its predictive value. It confirmed a strong correlation between psoriasis and both COPD and AS, demonstrating good predictive efficacy for COPD. Additionally, it clarified that factors such as age, smoking, and hypertension are associated with both airway diseases. This study not only addresses the limitations of previous research focusing on the association between psoriasis and a single airway disease but also provides potential evidence-based support for screening and precision management of comorbid risks in chronic airway diseases. It further lays the groundwork for subsequent exploration of common mechanisms among the three conditions and the development of cross-disease intervention strategies. However, the study is constrained by limitations in the NHIS database information. Future research should incorporate structured clinical indicators from electronic health records or conduct multicenter prospective studies for further validation.

## Supporting information

S1 Table Variable and its numbering information table. (DOCX)

S2 Table Results of multicollinearity analysis. (XLSX)

S1 Fig (A) Bar chart of missing rates for each variable, showing the distribution of missing data proportions across 27 key variables. The bar chart is ordered by missing rate from lowest to highest, intuitively presenting the data completeness of each variable. The specific missing percentage of each variable is labeled with numbers on the right side. (B) Statistical power plots for asthma data: the upper left subplot represents the statistical power distribution, the upper right subplot is a scatter plot showing the relationship between odds ratios (OR) and statistical power, and the lower subplot is a bar chart of the average statistical power for each effect. (C) Statistical power plots for COPD data.
(TIF)
