# Supplementary material for: Exploring the risk association between psoriasis and chronic obstructive pulmonary disease, and asthma using the NHIS database
Source: PLoS One. 2026 Feb 13;21(2):e0342015. doi: 10.1371/journal.pone.0342015 (PMC12904444; doi:10.1371/journal.pone.0342015)
Supplement: S1 Table — (DOCX) [file pone.0342015.s001.docx]

**S1 Table.** Variable and its numbering information table

| covariate | code | year |
| --- | --- | --- |
| age | AGEP_A | 2023 |
| gender | SEX_A | 2023 |
| race | HISDETP_A | 2023 |
| region | REGION | 2023 |
| education | EDUCP_A | 2023 |
| marital status | MARITAL_A | 2023 |
| employment | EMPLASTWK_A | 2023 |
| Income | RATCAT_A | 2023 |
| smoking | SMKCIGST_A | 2023 |
| BMI | BMICAT_A | 2023 |
| health | PHSTAT_A | 2023 |
| mental | DISAB3_A | 2023 |
| disability | MHTHRPY_A | 2023 |
| anxiety | ANXEV_A | 2023 |
| depression | DEPEV_A | 2023 |
| heart attack | MIEV_A | 2023 |
| coronary heart disease | CHDEV_A | 2023 |
| prediabetes | PREDIB_A | 2023 |
| hypertension | HYPEV_A | 2023 |
| high cholesterol | CHLEV_A | 2023 |
| cancer | CANEV_A | 2023 |
| arthritis | ARTHEV_A | 2023 |
| stroke | STREV_A | 2023 |
| health insurance | NOTCOV_A | 2023 |
