# Supplementary figures and images for: Exploring the risk association between psoriasis and chronic obstructive pulmonary disease, and asthma using the NHIS database
Source: PLoS One. 2026 Feb 13;21(2):e0342015. doi: 10.1371/journal.pone.0342015 (PMC12904444; doi:10.1371/journal.pone.0342015)

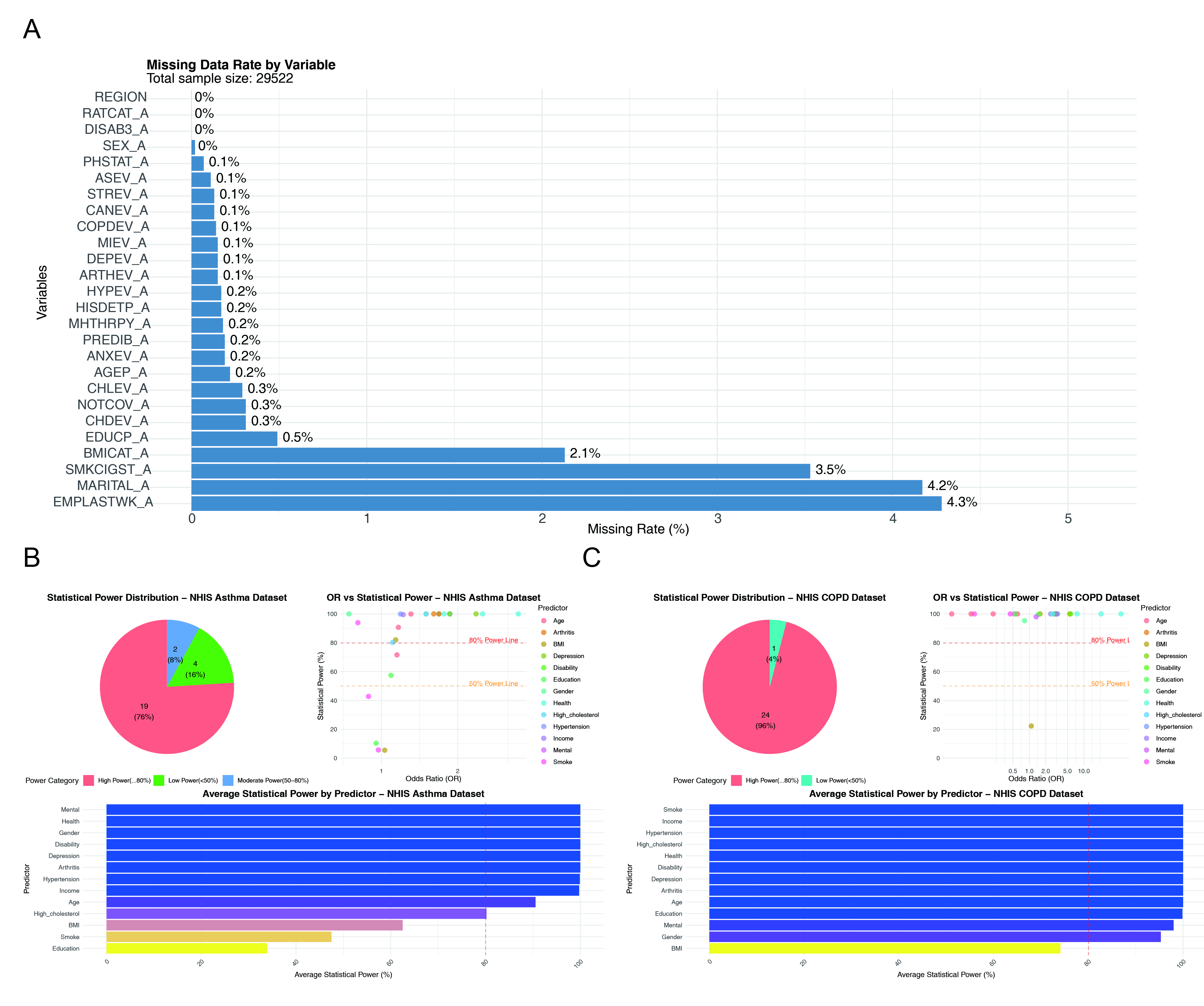

Supplement: S1 Fig — The bar chart is ordered by missing rate from lowest to highest, intuitively presenting the data completeness of each variable. The specific missing percentage of each variable is labeled with numbers on the right side. (B) Statistical power plots for asthma data: the upper left subplot represents the statistical power distribution, the upper right subplot is a scatter plot showing the relationship between odds ratios (OR) and statistical power, and the lower subplot is a bar chart of the average statistical power for each effect. (C) Statistical power plots for COPD data. (TIF) [file pone.0342015.s003.tif]
